# Small RNA sequencing identified miR-3180 as a potential prognostic biomarker for Chinese hepatocellular carcinoma patients

**DOI:** 10.3389/fgene.2023.1102171

**Published:** 2023-03-27

**Authors:** Libo Sun, Hansheng Zhou, Xiaofei Zhao, Haitao Zhang, Yan Wang, Guangming Li

**Affiliations:** ^1^ General Surgery Center, Beijing YouAn Hospital, Capital Medical University, Beijing, China; ^2^ Department of Pharmacy, Linyi People’s Hospital, Linyi, Shandong, China; ^3^ CAS Key Lab of Mental Health, Institute of Psychology, Beijing, China; ^4^ University of Chinese Academy of Sciences, Beijing, China

**Keywords:** miRNA, hepatocellular carcinoma, prognostic biomarker, overall survival, progression-free survival

## Abstract

MicroRNAs (miRNAs) and their target genes are aberrantly expressed in many cancers and are linked to carcinogenesis and metastasis, especially among hepatocellular carcinoma (HCC) patients. This study sought to identify new biomarkers related to HCC prognosis using small RNA sequencing from the tumor and matched normal adjacent tissue of 32 patients with HCC. Eight miRNAs were downregulated and 61 were upregulated more than twofold. Of these, five miRNAs, hsa-miR-3180, hsa-miR-5589-5p, hsa-miR-490-5p, hsa-miR-137, and hsa-miR-378i, were significantly associated with 5-year overall survival (OS) rates. Differential upregulation of hsa-miR-3180 and downregulation of hsa-miR-378i in tumor samples supported the finding that low and high concentrations of hsa-miR-3180 (*p* = 0.029) and hsa-miR-378i (*p* = 0.047), respectively, were associated with higher 5-year OS. Cox regression analyses indicated that hsa-miR-3180 (HR = 0.08; *p* = 0.013) and hsa-miR-378i (HR = 18.34; *p* = 0.045) were independent prognostic factors of poor survival. However, high hsa-miR-3180 expression obtained larger AUCs for OS and progression-free survival (PFS) and had better nomogram prediction than hsa-miR-378i. These findings indicate that hsa-miR-3180 may be associated with HCC progression and could serve as a potential biomarker for this disease.

## Introduction

Hepatocellular carcinoma (HCC) is the fifth most common of the major malignant liver tumors and the third leading cause of cancer-related mortality worldwide (Shariff et al., 2009; Guan et al., 2016). China has more than half (54%) of all HCC cases. Since liver cancer symptoms usually become evident at an advanced stage, only a minority of patients are eligible for curative resection (Wu et al., 2010). Liver cancer treatment remains unsatisfactory, reflecting the lack of understanding about liver carcinogenesis and the failure to develop interventions that block or reverse the process of malignant transformation. Thus, there is a need to improve patient care by identifying biomarkers capable of predicting treatment response.

MicroRNAs (miRNAs) are non-coding RNAs consisting of 21–23 nucleotides that modulate gene expression at a posttranscriptional level (Manikandan et al., 2008; Simon et al., 2008) and play a critical part in multiple biological processes, including cell formation, growth, apoptosis, and metastasis. MiRNA is stably expressed in plasma, serum, and other body fluids where they base pair to partially complementary mRNAs and degrade target mRNA transcripts or inhibit mRNA translation. Changes in miRNA expression are found to play an important role in different steps of tumor formation and progression (Calin and Croce, 2006), suggesting that they may serve as effective biomarkers for cancer diagnosis and prognosis (Simon et al., 2008; Zuberi et al., 2015; Armand-Labit and Pradines, 2017).

Studies have shown that miRNAs can function as tissue-specific biomarkers for liver cancer. The expression of many miRNAs is shown to be deregulated in human HCC tissue compared with normal tissue (Murakami et al., 2006; Ladeiro et al., 2008). While several studies have reported the potential diagnostic value of miRNAs in hepatocarcinogenesis (Negrini et al., 2007; Gailhouste and Ochiya, 2013), their value in HCC remains unclear. Current findings are conflicting or inconsistent due to differences in study design and specimen type. The current study sought to characterize the miRNA transcriptome in liver cancer and adjacent normal tissue from 32 primary HCC cases and analyze the expression of differentially expressed miRNAs to characterize the underlying molecular basis of HCC among northern Chinese patients.

## Methods

### Ethics

The use of tissues for this study has been approved by the Ethics Committee of the Beijing YouAn Hospital at Capital Medical University with approval number of LL-2016-060-K. At the time of initial diagnosis, all patients provided written informed consent to be involved in the study and for their tumor samples to be used for research purposes.

### Patient cohort and sample preparation

A total of 32 patients who were diagnosed with HCC and treated at the Beijing YouAn Hospital from January 2011 to June 2018 were included in this study. Clinical follow-up was received up to 5 years. Survival information was available for all patients. Resected specimens were processed routinely for histopathological assessment at the time of surgery and classified using the Tumor Node Metastasis (TNM) staging system. Additional tumor tissue was sampled by the surgeon after the specimen was removed from the patient, and immediately snap-frozen in liquid nitrogen. Samples were then transported to the research laboratory and kept for long-term storage at −80°C until RNA isolation.

### Total RNA extraction

Total RNA was extracted from the frozen tissues using the TIANamp DNA/RNA Isolation Kit (TIANGEN) and treated with RNase-free DNase I (Ambion) for 30 min at 37°C to remove contaminating DNA.

### MiRNA isolation and sequencing

RNA (1 µg) from the tumor and paired adjacent tissues of 32 HCC patients were used to generate a miRNA sequencing library. RNA segments (18–30 nt) were separated and recovered using a PAGE gel. The miRNA DNBs were loaded onto patterned nanoarrays and the 50 bp single-end reads were read using the BGISEQ-500 platform at Beijing Genomics Institute (BGI; Shenzhen, China).

### Sequencing data analysis

Raw sequencing reads were filtered with FASTQ to remove those that were low-quality, adapter contaminated, or shorter than 16 nt (Cock et al., 2010). Clean reads were mapped to the reference genome using anchor alignment-based small annotation (AASRA) (Tang et al., 2017). Those reads that matched with rRNAs and tRNAs were excluded. The remaining reads were aligned against the miRBase (v21) (Griffiths-Jones et al., 2008) using Bowtie and allowing for one mismatch. Unaligned sequences were pooled together to identify novel miRNAs with miRDeep (v2.0.0.5) software (An et al., 2013) using the default parameters. To determine the miRNA expression profiles, miRNA counts were normalized to tags per million (TPM) using the following formula: normalized expression = actual miRNA read count/total clean read count × 10^6^.

Differentially expressed miRNAs between the paired groups were analyzed using DEGseq (Wang et al., 2010). The *p*-values determined for each miRNA were adjusted to Q-values for multiple testing corrections using two alternative strategies (Storey and Tibshirani, 2003). To improve the overall accuracy of differentially expressed gene (DEG) results, a miRNA was defined as a differentially expressed miRNA (DE-miRNA) when Q-values were ≤0.05.

### MiRNA target prediction

Potential miRNA targets were identified using miRanda (v3.3a, parameters -en −20 -strict) (Enright et al., 2003; Betel et al., 2008) and TargetScan (v6.0, parameters -c 4) (Lewis et al., 2003). The sequences were defined as miRNA targets when predicted by both miRanda and TargetScan. If the number of target genes was <100, the top 100 were selected according to the scoring system. To reduce rate of false identification, the gene expression profile of putative targets had to correlate negatively with the miRNA profile.

### DAVID enrichment analysis

To determine the potential functions of the DE-miRNAs, a functional analysis of their target genes was performed using the web-based DAVID v6.8 tool (Huang da et al., 2009). Functional categories were clustered using the Functional Annotation Clustering tool and KEGG pathways from each clustered set with a *p*-value <0.05 were selected and considered for further analysis.

### Construction of a diagnostic nomogram

A clinical prediction nomogram to assess the risk of HCC was constructed using the R rms package (https://cran.r-project.org/web/packages/rms/index.html) with results from the final multivariable logistic regression (Nick and Hardin, 1999). Decision curve analysis (DCA) (Yu, 2018) was used to investigate the clinical utility of the nomogram by evaluating the net benefits at different threshold probabilities.

### Real-time PCR validation

Potential target genes expression of hsa-miR-3180 were validated with 32 pairs liver tissues by quantitative reverse-transcription PCR (RT-qPCR) using the Maxima SYBR Green qPCR Master Mix kit (Fermentas) according to the manufacturer’s instructions on an ABI Prism 7500 Sequence Detection System machine (Applied Biosystems Inc.). GAPDH was used as an internal control, and relative mRNA expression level was quantified using the 2^−ΔΔCt^ method. The qPCR primers used in this study were included in Supplementary Table S1.

### Statistical analysis

Hierarchical clustering was used to visualize the miRNA expression patterns. The association between miRNA expression and clinicopathological parameters was analyzed using a non-parametric test (Mann-Whitney U test between two groups and Kruskal–Wallis H test for ≥ three groups). Differences with a *p*-value <0.05 were considered statistically significant.

Survival curves were plotted using the Kaplan-Meier method (Lacny et al., 2018) and compared using the log-rank test. Survival data were evaluated using univariate and multivariate Cox regression analyses. In all cases, a *p* < 0.05 was considered statistically significant. A receiver-operator characteristic (ROC) curve was constructed and the area under the ROC curve (AUC) was determined by numerical integration. The TimeROC package was used to determine the maximum sensitivity and specificity of each miRNA needed to distinguish between different types of HCC, yielding corresponding optimal thresholds for defining miRNA levels (Blanche et al., 2013).

The expression values of qPCR were presented as means ± SEM. Statistical analysis of qPCR data was performed using a paired *t*-test with two tailed distributions using GraphPad Prism version 6.0. The results were considered statistically significant when *p* < 0.05.

## Results

### Patient characteristics

Patients with HCC who received medical surgery at YouAn Hospital in 2015 and 2017 were retrospectively selected. All 32 included cases underwent anatomic liver resection as previously described (Ruiz et al., 2016), and none had been treated with chemotherapy or radiation prior to tumor resection. Preoperative laboratory data were retrieved from all patients.

### MiRNA expression profile

HCC-associated miRNA biomarkers were identified through retrospective analysis of 32 prospectively collected tumor samples and matched normal tissue adjacent to the tumor (NAT) from HCC patients and assessed by age, sex, and ethnicity (Supplementary Table S2). There were around 20 million miRNA sequence reads required per sample library. The miRNA profiles of each sample were presented as transcripts per kilobase million (TPM).

**FIGURE 1:**
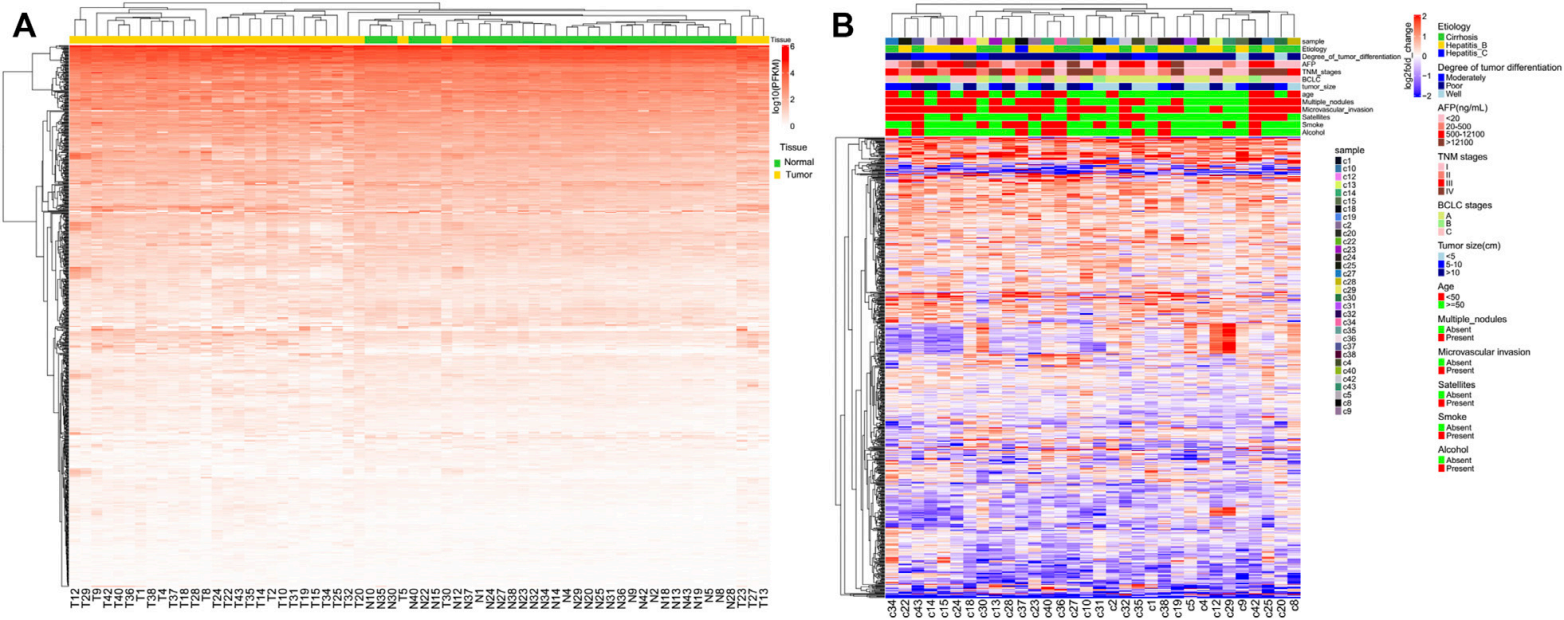
Unsupervised hierarchical clustering with complete linkage and Spearman correlation depicting **(A)** global miRNA expression in all HCC patients and **(B)** global log2fold_changes of each miRNA and each HCC patient.

Unsupervised hierarchical clustering was conducted using individual miRNAs from 32 clinical samples and matched adjacent NAT (Figure 1A). Most normal and tumor samples clustered together and showed obvious differences in miRNA expression. To better visualize these differences, log2fold_changes of each sample were transformed by DEseq2 for each miRNA. There was no obvious correlation between the differences in miRNA expression and patient characteristics (Figure 1B).

### Differential expressed miRNA analysis and target gene prediction

**FIGURE 2:**
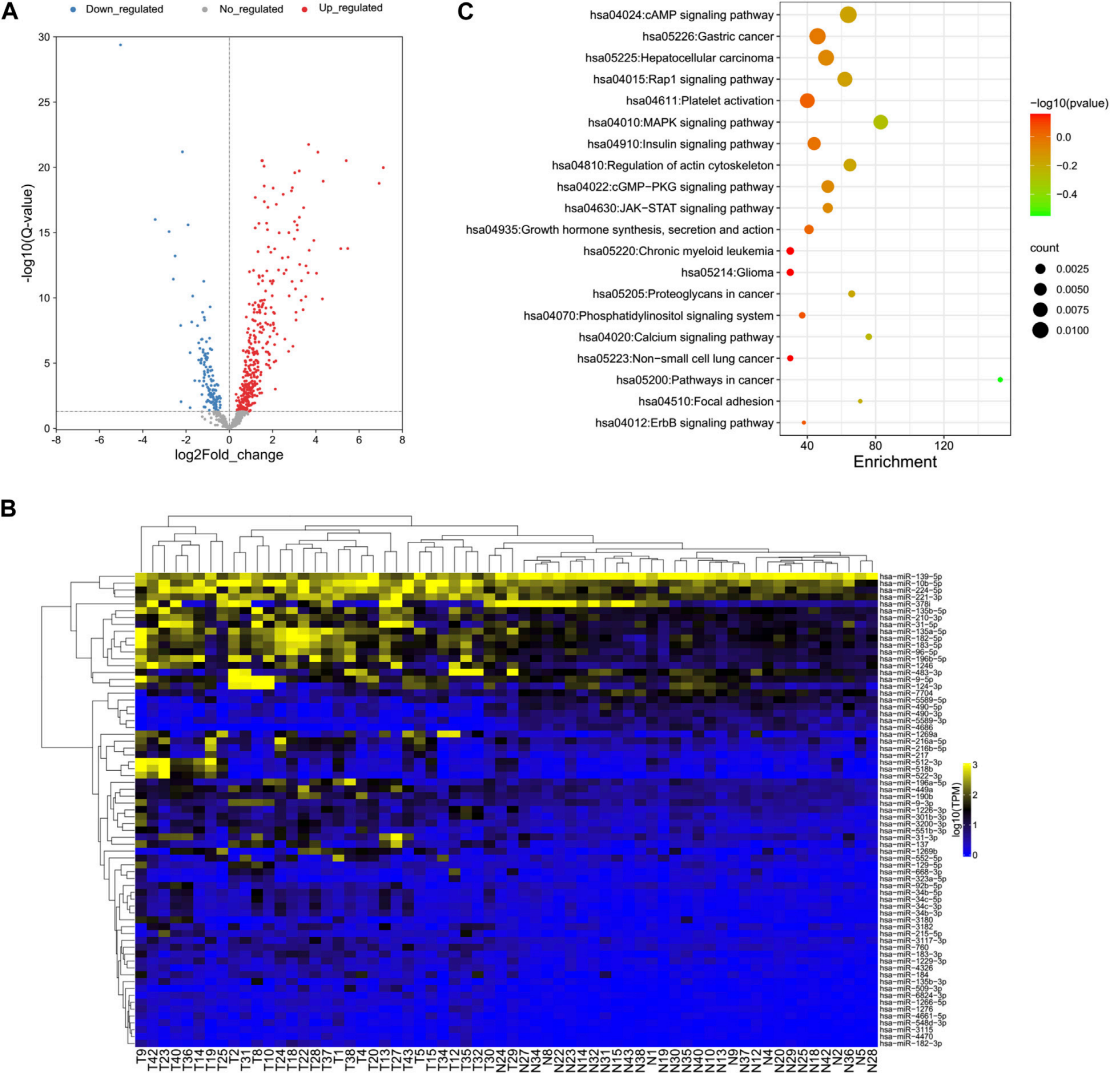
Global deep sequencing analysis of miRNAs from HCC tissue and normal adjacent tissue specimens. **(A)** Volcano plot of log2fold changes and -log10 (adjust *p*-value) differentially regulated miRNAs in HCC tissue and adjacent tissue specimens. Differentially expressed miRNAs were identified between all tumor and normal samples for each miRNA. **(B)** Unsupervised hierarchical clustering of differentially expressed miRNA expression from all patients. **(C)** Chart of top 20 enriched pathways of the target genes of differentially expressed miRNAs predicted by miRanda and TargetScan.

DEseq2 was used to identify the DE-miRNAs between all tumor and normal samples for each miRNA (Love et al., 2014). A total of 138 miRNAs were downregulated and 348 miRNAs were upregulated in tumor versus adjacent normal tissue (Figure 2A). Cluster analysis based on the DE-miRNAs was used to generate a phylogenetic tree and showed a clear distinction between cancer and normal tissues (Figure 2B). Eight miRNAs were upregulated and 61 miRNAs were downregulated by more than twofold in HCC tissue compared with NAT (Supplementary Table S3). The top five downregulated miRNAs in HCC samples were hsa-miR-4686, hsa-miR-490-5p, hsa-miR-5589-3p, hsa-miR-490-3p, and hsa-miR-7704, while the top five upregulated miRNAs were hsa-miR-1269a, hsa-miR-137, hsa-miR-522-3p, hsa-miR-518b, and hsa-miR-512-3p.

Since the biological significance of miRNA deregulation is reliant on how their cognate protein-coding gene targets are affected, the predicted targets of the significantly up- and downregulated miRNAs were assessed. The analysis was performed using miRanda and TargetScan, which are commonly used to predict human miRNA gene targets. The intersection of prediction from these programs was used to identify a final total of 4,313 target genes (Supplementary Table S4). KEGG pathway analysis revealed that almost half of the top 20 enriched pathways were cancer-related pathways, which is reasonable for the liver cancer samples and proved the accuracy of our analysis results (Figure 2C).

### Survival analysis of differentially expressed miRNAs

**FIGURE 3:**
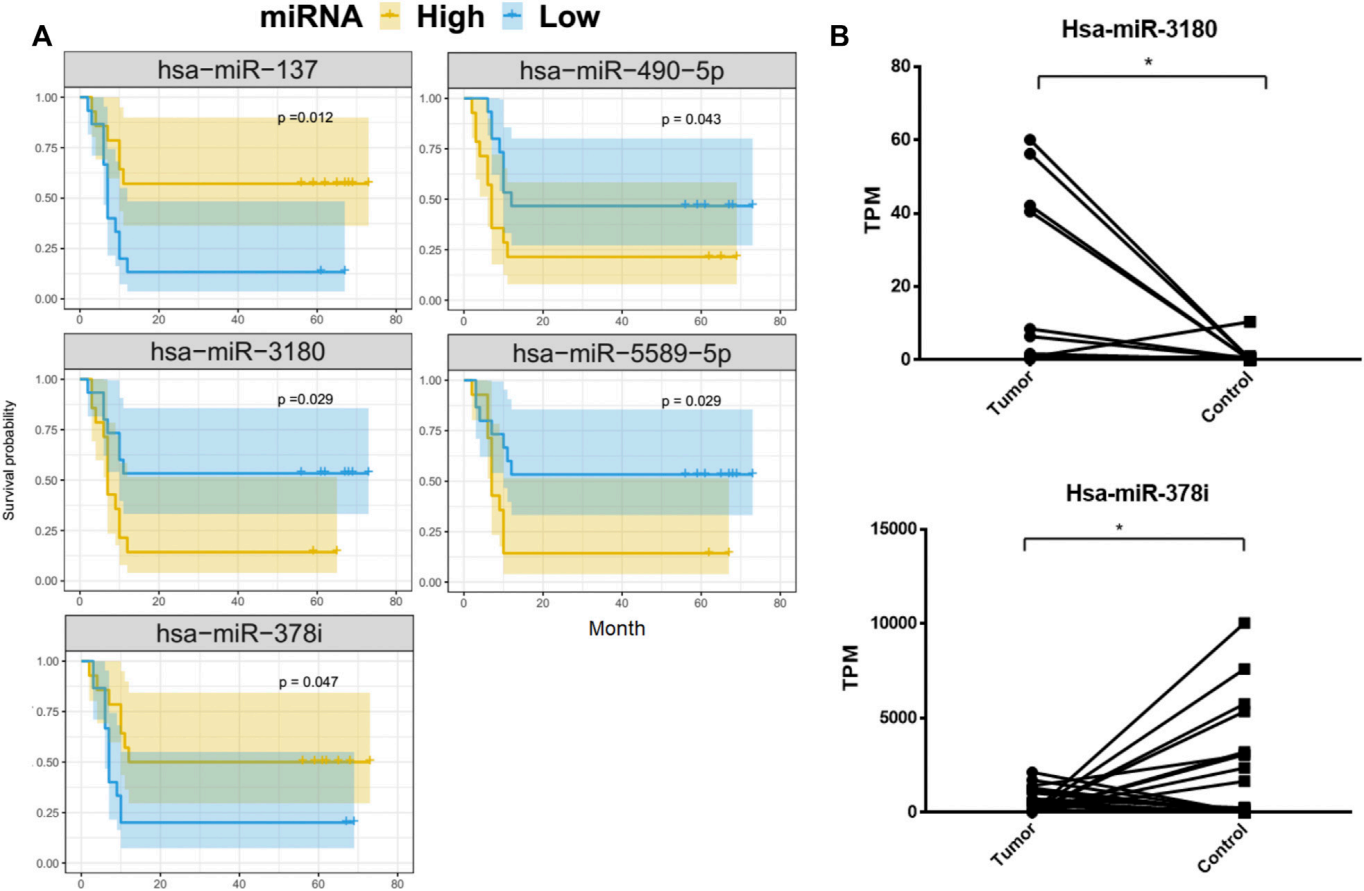
Kaplan-Meier overall survival curves for 32 HCC patients. Tumor miRNA expression was divided into two groups and the log-rank test was used to compare the survival curves of the high- and low-expression groups. **(A)** MiRNAs that were significantly associated with overall survival. **(B)** Differences in the expression of hsa-miRNA-3180 and hsa-miRNA-378i between HCC and adjacent normal liver tissue from 32 patients.

To better understand the relationship between the 69 DE-miRNAs and patient survival, 32 HCC patients were enrolled with complete follow-up data. Using Kaplan-Meier analysis, hsa-miR-137, hsa-miR-3180, hsa-miR-378i, hsa-miR-490-5p and hsa-miR-5589-5p were shown to be associated with HCC patient prognosis and disease progression. The remaining miRNAs were not significantly predictive of HCC patient outcomes (Figure 3A).

HCC patients with low concentrations of hsa-miR-3180, hsa-miR-490-5p, and hsa-miR-5589-5p had higher 5-year OS rates than those with high concentrations (*p* = 0.029, *p* = 0.043, *p* = 0.029, respectively). In contrast, HCC patients with high concentrations of hsa-miR-137 and hsa-miR-378i had higher 5-year OS rates than those with low concentrations (*p* = 0.012 and *p* = 0.047, respectively). Of these, hsa-miR-3180 was upregulated in tumor samples and hsa-miR-378i was downregulated, indicating that changes in the expression in these miRNAs reduced 5-year OS rates (Figure 3B).

### Cox regression analysis of miRNAs

To explore the association between the five survival-associated miRNAs, hsa-miR-137, hsa-miR-3180, hsa-miR-378i, has-miR-490-5p and hsa-miR-5589-5p, and 12 prognostic-related clinical characteristics, including AFP (Alpha Fetoprotein), BCLC stage (Barcelona clinic liver cancer stage), degree of tumor differentiation, microvascular invasion, multiple nodules, satellites, TNM stage (The tumor-node-metastasis stage), tumor size, age, alcohol use, etiology, sex and smoking on the OS of HCC patients (Table 1), Cox regression analysis was used to identify factors associated with PFS (progression-free survival). Univariate Cox regression analysis showed that AFP, BCLC stage, degree of tumor differentiation, microvascular invasion, multiple nodules, satellites, TNM stages, tumor size, and hsa-miR-137, hsa-miR-3180, hsa-miR-378i, and hsa-miR-5589-5p expression correlated significantly with HCC prognosis (*p* < 0.05).

**TABLE 1:** Univariate and multivariate Cox proportional hazard models for the OS of 32 HCC patients by clinical and demographic factors and miRNAs.

	Univariate cox			Multivariate cox		
Characteristics	Hazard ratio	CI95	*p*-value	Hazard ratio	CI95	*p*-value
AFP	1.99	1.08–3.66	0.027	0.63	0.2–1.98	0.426
Age	1.16	0.46–2.88	0.756			
Alcohol	2.38	0.89–6.37	0.085			
BCLC stage	2.03	1.19–3.47	0.01	0.9	0.29–2.74	0.849
Degree of tumor differentiation	3.53	1.4–8.89	0.008	1.72	0.47–6.32	0.411
Etiology	0.93	0.37–2.31	0.872			
Microvascular invasion	5.75	1.3–25.35	0.021	0.65	0.05–7.93	0.738
Multiple nodules	6.59	2.08–20.93	0.001	5.81	0.46–73.55	0.174
Satellite	3.38	1.32–8.6	0.011	0.72	0.17–3	0.65
Sex	0.61	0.2–1.85	0.38			
Smoke	0.92	0.35–2.41	0.858			
TNM stage	2.13	1.36–3.35	0.001	1.22	0.41–3.62	0.721
Tumor size	3.61	1.42–9.21	0.007	3.57	0.69–18.48	0.129
hsa-miR-137	3.37	1.26–9.02	0.016	1.06	0.03–34.85	0.973
hsa-miR-3180	0.36	0.14–0.93	0.035	0.08	0.01–0.59	**0.013**
hsa-miR-378i	2.62	1.01–6.77	0.048	18.34	0.84–40.56	**0.045**
hsa-miR-490-5p	0.4	0.16–1.01	0.053			
hsa-miR-5589-5p	0.34	0.13–0.89	0.028	0.96	0.16–5.57	0.96

Multivariate Cox proportional hazards models were also used to identify independent predictors of PFS. Only hsa-miR-3180 and hsa-miR-378i were independently associated with the OS of HCC patients (*p* = 0.013 and *p* = 0.045, respectively, bold words in the Table 1) suggesting that these miRNAs play and important role in hepatocellular carcinogenesis.

### Constructing and assessing a nomogram

**FIGURE 4:**
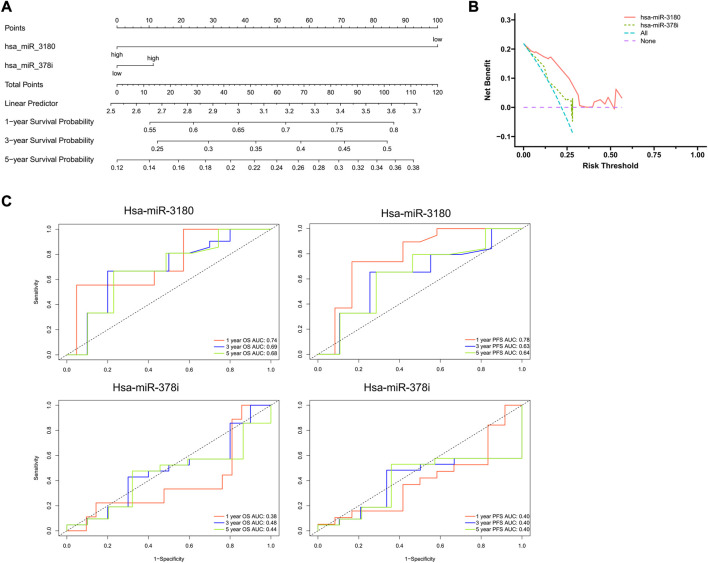
Nomogram construction and evaluation. **(A)** Nomograms based on hsa-miRNA-3180 and hsa-miRNA-378i were used to predict the probabilities of 3- and 5-year OS. **(B)** Decision curve analysis for the nomogram, hsa-miR-3180, and hsa-miR-378i. The x-axis shows the threshold probability, and the y-axis measures the net benefit. The red line represents the model with hsa-miR-3180. The olive line represents the model with age hsa-miR-378i. **(C)** Time-dependent ROC curve analyses of the sensitivity and specificity of hsa-miRNA-3180 and hsa-miRNA-378i for 1-, 3-, and 5-year survival. OS: overall survival; ROC curve: receiver operating characteristic curve.

Using results from the final multivariate logistic regression, a nomogram was established with two indicator-based miRNAs to predict 1-, 3- and 5-year survival (Figure 4A). Each of the miRNAs corresponded vertically to the points, and the sum was considered the total number of points. Calibration curves found that the nomogram was relatively well-calibrated and capable of adequate prediction (Figure 4B).

A ROC (receiver operating characteristic) curve was created to evaluate the predictive efficiency of the nomogram. Time-dependent ROC curve analysis was used to predict 1-, 3- and 5- year PFS and OS. Hsa-miR-3180 had larger AUCs for PFS and OS at all follow-up time points (0.78 and 0.74 at 1-year of PFS and OS, respectively, for hsa-miR-3180; 0.38 and 0.40 for 1-year of PFS and OS, respectively, for hsa-miR-378i). These results indicated that hsa-miR-3180 could serve as a biomarker for HCC (Figure 4C).

### Correlation between miRNA expression and the clinicopathological characteristics of HCC patients

**FIGURE 5:**
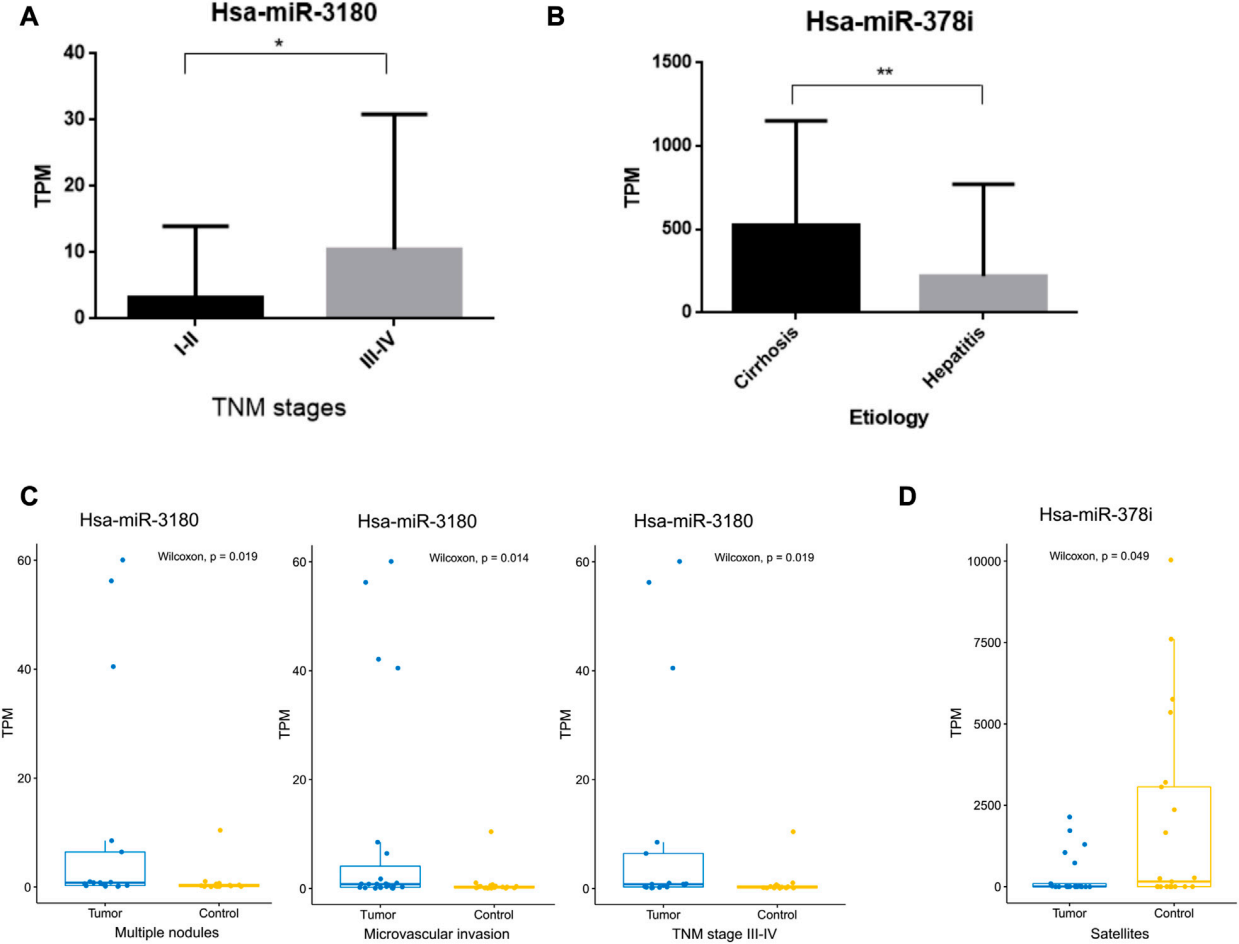
Correlation between hsa-miRNA-3180 and hsa-miRNA-378i and clinical pathological parameters. **(A)** Significant differences in hsa-miRNA-3180 expression between the early (I-II) and late (III-IV) TNM stages of HCC. **(B)** Significant differences in hsa-miRNA-378i expression between cirrhosis and hepatitis. **(C)** Significant changes in hsa-miRNA-3180 expression by TNM stage (III-IV) and the presence of multiple nodes, microvascular invasion, and satellites between the tumor and adjacent normal liver tissues from 32 HCC patients.

The clinical significance of hsa-miR-3180 and hsa-miR-378i was determined by measuring their associations with clinicopathological characteristics. High expression of hsa-miR-3180 correlated with TNM stage (*p* = 0.049) (Table 2; Figure 5A). Other parameters such as etiology, degree of tumor differentiation, microvascular invasion, AFP, BCLC stage, tumor size, multiple nodules, and satellites did not correlate significantly with high hsa-miR-3180 expression. Meanwhile, there was a significant difference in hsa-miR-3180 expression in tumors that were TNM stage III–IV (*p* = 0.018), had multiple nodules (*p* = 0.023), or had microvascular invasion (*p* = 0.014) (Figure 5C).

**TABLE 2:** Clinicopathological patient characteristics and hsa-miR-3180 and hsa-miR-378i expression in HCC tissues.

Characteristics	Num	hsa-miR-3180		hsa-miR-378i	
		*p*	*p* (Tum vs. Nor)	*p*	*p* (Tum vs. Nor)
**Age**		0.35		0.89	
≥50	20		0.093		0.38
<50	12		0.038		0.30
**Etiology**		0.17		**0.007**	
Cirrhosis	14		0.053		0.26
Hepatitis B & C	18		0.1098		0.013
**TNM stage**		**0.049**		0.94	
Stage I-II	15		0.36		0.27
Stage III- IV	17		0.018		0.36
**Degree of tumor differentiation**		0.87		0.41	
Poor	22		0.095		0.052
Moderate & Well	10		0.089		0.52
**Multiple nodules**		0.22		0.78	
Absent	14		0.35		0.16
Present	18		0.023		0.48
**Microvascular invasion**		0.089		0.65	
Absent	9		0.30		0.22
Present	23		0.014		0.41
**Tumor size(cm)**		0.52		0.054	
<5	12		0.18		0.11
5–10	9		0.17		0.23
≥10	11		0.12		0.22
**BCLC stage**		0.49		0.65	
A	14		0.11		0.57
B	6		0.23		0.24
C	12		0.25		0.48
**AFP (ng/mL)**		0.12		0.058	
<20	12		0.72		0.84
20–500	6		0.065		0.82
500–121000	10		0.021		0.12
>121000	4		0.40		0.49
**Satellite**		0.42		0.69	
Absent	21		0.062		0.049
Present	11		0.11		0.84

Significant *p* values of different stages of clinical factors were in bold.

Low expression of hsa-miR-378i also correlated with etiology (*p* = 0.007) (Table 2; Figure 5B). A significant difference in hsa-miR-378i expression was associated with hepatitis B and C etiology stage (Figure 5D). These results suggest that hsa-miR-3180 has a stronger association with HCC patient clinicopathological characteristics than hsa-miR-378i, and confirms the high potential of hsa-miR-3180 to serve as a biomarker for HCC diagnosis.

### hsa-miR-3180 target genes and their biological function

**FIGURE 6:**
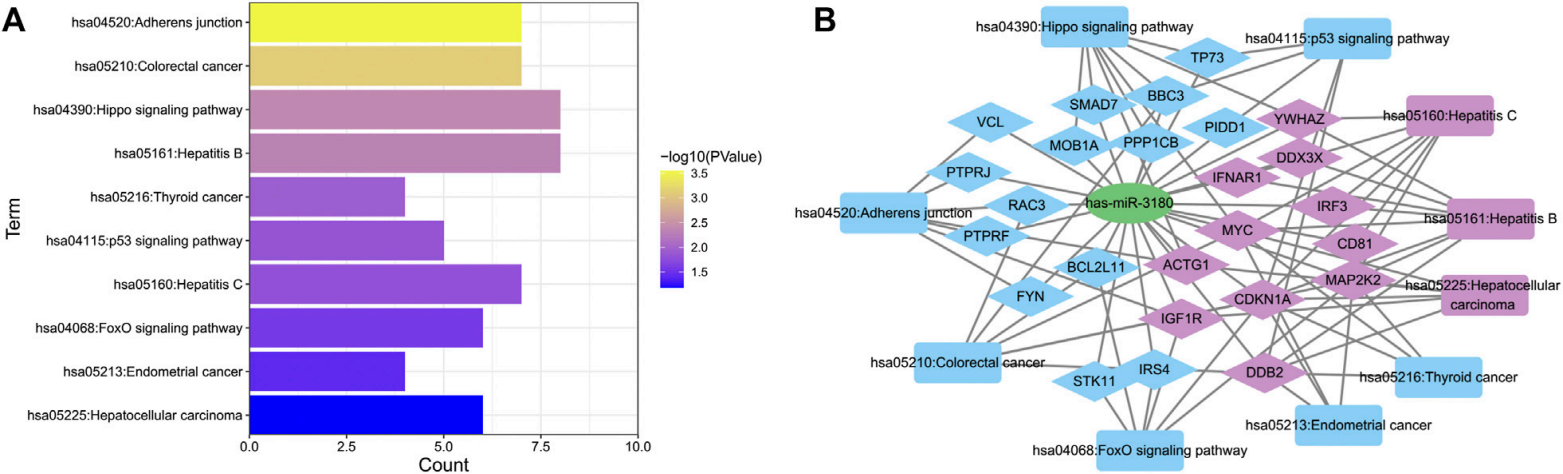
Biological function of target genes of hsa-miRNA-3180. **(A)** Chart of top 10 enriched pathways of the target genes of hsa-miRNA-3180. **(B)** Network of hsa-miRNA-3180 and its target genes and enriched pathways. Purple represents target genes and pathways related with hepatitis and liver carcinoma and blue represents target genes and pathways related with other pathways.

To further elucidate the biological function of hsa-miR-3180, we predicted targeted gene and performed biological enrichment analysis using the database for annotation, visualization, and integrated discovery (DAVID) KEGG terms. The top 10 pathways were enriched in cancer related pathways such as Colorectal cancer pathway, Thyroid cancer and Endometrial cancer, and liver diseases related pathways such as Hepatitis B, Hepatitis C and Hepatocellular carcinoma pathways (Figure 6A). Meanwhile, target genes of hsa-miR-3180 enriched in top 10 KEGG pathways were visualized with Cytoscape_3.9 (Figure 6B). Almost half of these genes (purple-colored genes) were enriched in Hepatitis B, Hepatitis C and Hepatocellular carcinoma pathways including MYC, CD81, CDKN1A, IGF1R and so on (Lin et al., 2010; Li et al., 2020; Ngo et al., 2021) which were reported in Hepatocellular carcinoma. These suggested that hsa-miR-3180 plays an important role in the liver carcinogenesis by regulating target genes.

### Validation of hsa-miR-3180 expression in TCGA and SRA cohort

**FIGURE 7:**
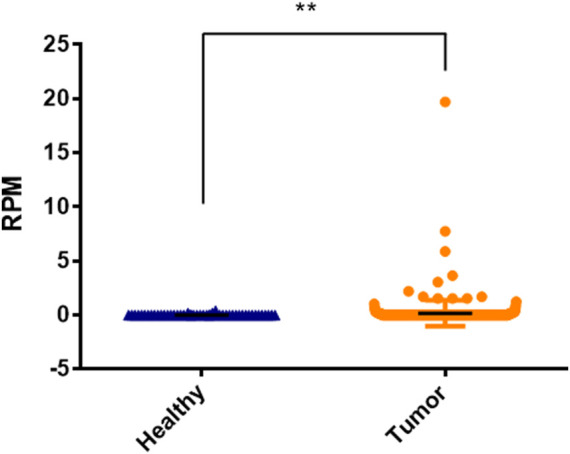
Significantly expressed differences of hsa-miRNA-3180 between healthy and disease samples of hepatocellular cancer.

We used TCGA cohort to externally verify the expression of hsa-miR-3180 in HCC based on DIANA-miTED database which was collected from NCBI-SRA and TCGA analyzed datasets (Kavakiotis et al., 2022). Expression of hsa-miR-3180 was extracted from tumor samples and healthy samples of hepatocellular cancer. *t*-test analysis showed that there is a significantly higher expression of hsa-miR-3180 in tumor samples than in healthy samples (*p* = 0.013, Figure 7). This was consistent with our results of hsa-miR-3180, confirmed the clinical relevance of hsa-miR-3180 in HCC.

### Validation of potential target genes of hsa-miR-3180

**FIGURE 8:**
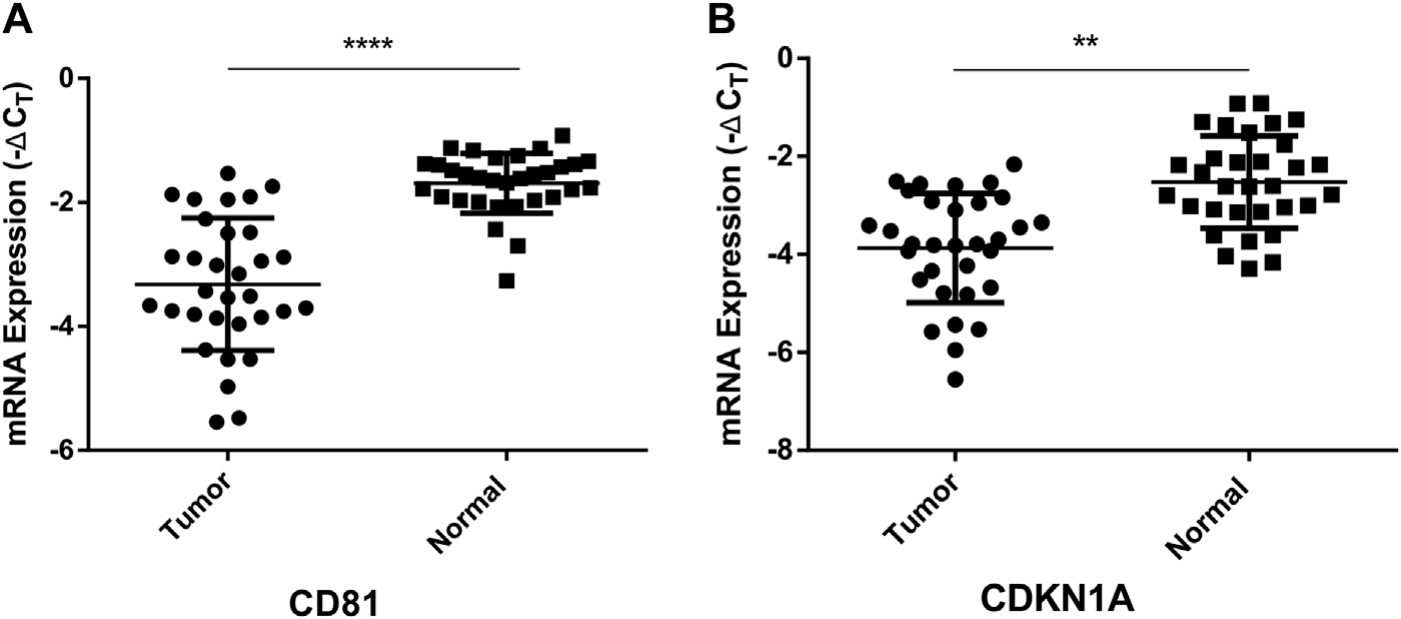
Quantitative real-time PCR analysis of the relative mRNA expression levels of CD81 **(A)** and CDKN1A **(B)** in tumor tissues as compared to adjacent normal tissues. **p < 0.01, ****p < 0.0001.

CD81(CD81 Molecule) and CDKN1A (Cyclin Dependent Kinase Inhibitor 1A), the potential target genes of hsa-miR-3180, were located in liver diseases pathways (Figure 6). It is well known that low expression of CD81 has more metastatic potential in HCC cell lines and the CDKN1A suppression facilitates cell cycle progression from the G1 to the S phase to promote tumor cell proliferation (Mazzocca et al., 2008; Di Giorgio et al., 2015). The RT-qPCR assay results illustrated that both CD81 and CDKN1A were significantly downregulated in the tumor tissue compared adjacent normal tissue (*p* < 0.0001 of CD81 and *p* = 0.0029 of CDKN1A, Figure 8). This suggested that higher expressed hsa-miR-3180 may promote carcinogenesis and invasion through inhibited CD81 and CDKN1A expression.

## Discussion

The incidence of HCC has gradually increased in China and is attracting increasing attention from researchers and clinicians (Seo et al., 2016). HCC is often diagnosed in the mid and late stages of the disease when patients have missed the opportunity for successful surgical resection, the prognosis is poor due to rapid tumor growth, and there is a higher likelihood of metastasis (Mak et al., 2018). The current study sought to identify miRNAs that may serve as disease markers for the early detection of HCC.

HCC miRNA sequencing analysis identified 138 and 348 miRNAs that were downregulated and upregulated, respectively, compared to normal adjacent tissue. Another recent study of HCC identified a higher number of downregulated than upregulated miRNAs in tumor samples (Zhu et al., 2021). This may due to the different patient regions and different tumor locations. In the current study, eight miRNAs, including hsa-miR-4686, hsa-miR-490-5p, hsa-miR-5589-3p, hsa-miR-490-3p, and hsa-miR-7704, were downregulated more than twofold, and 61 miRNAs, including hsa-miR-1269a, hsa-miR-137, hsa-miR-522-3p, hsa-miR-518b, and hsa-miR-512-3p, were upregulated more than twofold. While some of these miRNAs have been reported previously, studies on miRNA expression in HCC tissues have yielded variable results (Zhang et al., 2020). The findings reported here provide additional insight into the functional and mechanistic role of miRNAs in HCC carcinogenesis.

Five miRNAs were significantly associated with 5-year OS rates among patients with HCC. High levels of hsa-miR-3180, hsa-miR-5589-5p, and hsa-miR-490-5p and lower levels of hsa-miR-137 and hsa-miR-378i correlated with poor survival outcomes. Several studies have shown that low hsa-miR-490 expression is associated with reduced HCC patient survival (Wang et al., 2018; Yang et al., 2018). However, miR-490 expression has also been positively correlated with OS of patients with HCC, lung cancer, and multiple myeloma (MM) (Zhang et al., 2013; Li et al., 2016; Lu et al., 2021). This may be due to the dual role of miR-490-5p in cancer regulation (Li et al., 2021).

Logistic regression with penalized estimates can be used to develop prognostic models for binary outcomes, especially when limited data are available. In the current study, univariate and multifactorial Cox regression analyses found that only hsa-miR-3180 (HR = 0.08; *p* = 0.013) and hsa-miR-378i (HR = 18.34; *p* = 0.045) were independent prognostic factors for the poor survival of patients with HCC. While hsa-miR-3180 was upregulated in tumor samples, hsa-miR-378i was downregulated, indicating that changes in the expression of these miRNAs reduced 5-year OS rates. Importantly, a dramatic reduction in expression of the miR-378 family is also shown in Rhabdomyosarcoma tumor tissue (Megiorni et al., 2014).

In the current study, hsa-miR-3180 was associated with larger areas under the ROC curve for PFS and OS after 1, 3, and 5 years of follow-up than hsa-miR-378i. The survival nomogram incorporating hsa-miR-3180 had an enhanced ability to predict patient survival than hsa-miR-378i. Meanwhile, hsa-miR-3180 expression was significantly higher in tumor tissue than in NAT and was associated with poor survival and high TNM stage. In addition, hsa-miR-3180 was significantly higher in the tumors of patients >50 years of age, TNM stage III-IV tumors, and those with multiple nodules, suggesting that hsa-miR-3180 can serve as a potent prognostic marker in HCC patients.

Hsa-miR-3180 has been reported upregulated and correlates with a higher TNM stage in the human gastric carcinoma cell line, MGC-803 (Wang et al., 2020), similar with the result we got. Sun et al. (2016) found that the bladder smooth muscle cell viability was markedly increased in the miR-3180 mimics group and markedly attenuated in the miR-3180 inhibitor group. In this paper, both our results and the public data showed the hsa-miR-3180 is overexpressed in HCC and the predicted target genes enriched in Hepatitis B, Hepatitis C and Hepatocellular carcinoma pathways indicating hsa-miR-3180 plays an important role in the liver carcinogenesis. While, the function of miR-3180 in HCC is still unknown. A full understanding of miR-3180 remains limited and further study is needed to characterize its function and mechanism in HCC.

## Data Availability

The data presented in the study are deposited in the GEO repository, accession number GSE227378.

## Abbreviations

HCC, hepatocellular carcinoma; OS, overall survival; PFS, progression-free survival; ROC, receiver operating characteristic; AUC, area under the curve; PAGE, gel, polyacrylamide gel electrophoresis gel; DNB, DNA, nanoball; TPM, tags per million; DCA, decision curve analysis; NAT, tissue adjacent to the tumor; TNM, stage, The tumor-node-metastasis stage; AFP, alpha fetoprotein; BCLC, stage, Barcelona clinic liver cancer stage.
