# Supplementary material for: Small RNA sequencing identified miR-3180 as a potential prognostic biomarker for Chinese hepatocellular carcinoma patients
Source: Front Genet. 2023 Mar 27;14:1102171. doi: 10.3389/fgene.2023.1102171 (PMC10083302; doi:10.3389/fgene.2023.1102171)
Supplement: Supplementary file 3 [file Table1.DOCX]

Table_S1: The primer sequence for each validated gene.

| **Primer name** | **Primer sequence (5′-3′)** |
| --- | --- |
| GAPDH- FW | TGACAACTTTGGTATCGTGGAAGG |
| GAPDH-RV | AGGCAGGGATGATGTTCTGGAGAG |
| CDKN1A-FW | GGGATGTCCGTCAGAACCCA |
| CDKN1A-RV | CACCCTCCAGTGGTGTCTCG |
| CD81-FW | GAGTGGAGGGCTGCACCAAGT |
| CD81-RV | CATCCACCACGGCCTGCTGTA |

Table_S2: Clinical features of 32 HCC patients (see excel table_s1).

Table_S3: miRNAs differentially expressed greater than twofold in HCC compared with NAT (see excel table_s2).

Table_S4: predicted target mRNAs of miRNAs differentially expressed greater than twofold (see excel table_s3).
